# Peer engagement in harm reduction strategies and services: a critical case study and evaluation framework from British Columbia, Canada

**DOI:** 10.1186/s12889-016-3136-4

**Published:** 2016-05-27

**Authors:** Alissa M. Greer, Serena A. Luchenski, Ashraf A. Amlani, Katie Lacroix, Charlene Burmeister, Jane A. Buxton

**Affiliations:** BC Centre for Disease Control, 655 West 12th Avenue, Vancouver, British Columbia V5Z 4R4 Canada; School of Population and Public Health, University of British Columbia, 2206 East Mall, Vancouver, BC V6T 1Z3 Canada; The Farr Institute of Health Informatics Research, University College London, 222 Euston Road, London, NW1 2DA United Kingdom; Society of Living Illicit Drug Users, 857 Caledonia Street, Victoria, British Columbia V8T 1E6 Canada

**Keywords:** Peer engagement, Community engagement, Public participation, Substance use, Harm reduction, Health equity, Process evaluation

## Abstract

**Background:**

Engaging people with drug use experience, or *‘peers,*’ in decision-making helps to ensure harm reduction services reflect current need. There is little published on the implementation, evaluation, and effectiveness of meaningful peer engagement. This paper aims to describe and evaluate peer engagement in British Columbia from 2010–2014.

**Methods:**

A process evaluation framework specific to peer engagement was developed and used to assess progress made, lessons learned, and future opportunities under four domains: supportive environment, equitable participation, capacity building and empowerment, and improved programming and policy. The evaluation was conducted by reviewing primary and secondary qualitative data including focus groups, formal documents, and meeting minutes.

**Results:**

Peer engagement was an iterative process that increased and improved over time as a consequence of reflexive learning. Practical ways to develop trust, redress power imbalances, and improve relationships were crosscutting themes. Lack of support, coordination, and building on existing capacity were factors that could undermine peer engagement. Peers involved across the province reviewed and provided feedback on these results.

**Conclusion:**

Recommendations from this evaluation can be applied to other peer engagement initiatives in decision-making settings to improve relationships between peers and professionals and to ensure programs and policies are relevant and equitable.

## Background

People who use illicit drugs are more likely to contract HIV and hepatitis C virus [1], experience mental and physical illness [2], and die prematurely [3]. The consequences of drug use negatively impact individuals, families, communities and society as a whole [3, 4]. Harm reduction is internationally recognized as best practice to prevent the transmission of blood-borne infections, promote safer drug use and safer sexual behaviours, increase access to social services and supports, and prevent and reverse overdoses [5]. However, simply making no-cost supplies and services available is not sufficient for providing comprehensive harm reduction interventions; services must also be accessible, accommodating, affordable, and acceptable (i.e. equitable regardless of drug used, route of administration, or where reside) [6].

Engaging people who use or have used drugs, *herein referred to as ‘peers’,* to participate in policy making, research, programming, and practice is fundamental to harm reduction globally [7]. The definition of ‘peers’ varies across the literature, but can be defined as “any persons with equal standing within a particular community who share a common lived experience” [7]. ‘Peers’ in the context of harm reduction are “people with lived experience of drug use work both behind the scenes and at the forefront of needle distribution services, harm reduction education, peer support, and community-based research initiatives” [8], providing valuable insights about the barriers and facilitators to accessing harm reduction services in their communities. Peer roles can be considered across multiple dimensions, including political advocacy, research assistance, program governance, peer support, and harm reduction messaging [8].

Peer engagement has been defined as a community-based approach to decision making by “consulting and collaborating with decision makers using a bottom-up approach in order to better address the needs of the community” [7]. Methods applied to engaging peers can vary considerably. Several frameworks for engagement and participation have been developed for examining citizen participation, although none of these models pertain specifically to peers. Arnstein’s ‘ladder of citizen participation’ was first published over forty years ago [9]; since, adapted versions have emerged including Hart’s ladder of youth participation [10] and Pretty’s participatory learning model for sustainability [11]. In all models, a policy, program or project can elicit equitable participation in resources, recognition, results, and knowledge by sharing power in partnerships [12]. Many peer engagement efforts are limited to exchanging information without sharing any decision-making authority; thus, efforts are merely “*tokenism”* [9]. Peers are increasingly involved in varying roles, but still underutilized, in the prevention of substance use related harms [8]. In 2013, a national symposium of fourteen peer-run organizations across Canada concluded that “tokenism and lack of representation are still common” obstacles to meaningful participation [13]. Stigma and discrimination also make it more difficult for peers becoming engaged in decision making processes [7].

### Peer engagement in British Columbia

Harm reduction efforts in the Canadian province of British Columbia (BC) provide a case study of where community-based engagement with people who use drugs – peers – has gained momentum. Since 2003, the BC Centre for Disease Control (BCCDC) oversees and coordinates provincial distribution of harm reduction efforts including safer drug use and sex supplies [14]. As part of the BCCDC, the BC Harm Reduction Services and Strategies (BCHRSS) committee guide provincial harm reduction policies and convene quarterly, alternating between in-person and teleconference meetings. The committee includes representatives from the BC Ministry of Health, five regional Health Authorities, and First Nations Healthy Authority [15].

In 2007, an increase in peer engagement in BC coincided [6] with the release of *“Nothing About Us Without Us,”* a report published by the Canadian HIV/AIDS Legal Network which makes a compelling case for meaningful peer involvement from a public health, ethical, and human rights perspective [16]. From this report, the BCHRSS committee agreed: “people who use illegal drugs should be engaged in all aspects of harm reduction supply distribution program development, implementation, and evaluation” [6], and in 2008 increased support for peers by contributing to several peer-run events, including financial support for a conference organized by a peer-run group, Vancouver Area Network of Drug Users (VANDU) [17]. Thereafter, efforts to develop and expand meaningful peer engagement have increased and the BCHRSS committee has officially embraced peer engagement as an essential first step in decision-making.

Peer engagement has the potential to augment equity of harm reduction services by fostering communication, building trust, increasing knowledge, and reducing stigma and discrimination to remove barriers and increase utilization of harm reduction services; this, in turn, will have a direct impact on mental and physical health. Globally, public health research and practice has shown that involvement of people with lived experience results in improved health outcomes and reduced health disparities by improving the acceptability and utilization of health services and removing barriers to access [7]. However, despite the increasing international support for engaging various populations [7, 8], there is very little published evidence on the implementation, evaluation, or effectiveness of meaningful engagement with peers. This paper aims to describe and evaluate the peer engagement efforts undertaken by the BCHRSS committee from 2010 to 2014. We highlight key lessons learned and improvements needed to ensure meaningful peer engagement in the planning, delivery and evaluation of harm reduction efforts.

## Methods

### Evaluation resources and appraisal

The evaluation team appraised the events, processes and products of peer engagement in the harm reduction program from January 2010 – December 2014 using a peer engagement evaluation framework developed specifically for this project (described below). The evaluation team included BCCDC staff as well as two peers. The peers involved in the evaluation had participated in BCHRSS meetings and other peer engagement activities with health authorities between 2010 and 2014. Evidence was obtained through a desk-based document review of BCHRSS materials. The document review was a retrospective review and synthesis of all relevant documents concerning the state of policies and provision of services and programs to identify social impacts and priorities for action [18]. One research assistant (SL) did the initial gathering of documents and literature; however, the findings were reviewed and discussed with all authors, including peers (CB & KL), and the drafting of this manuscript was in partnership with peers. The lack of more collaborative involvement of the gathering of information was mainly due to the geographic distance between team members.

Materials included formal documents, notes from relevant previously conducted focus groups and interviews, meeting agendas, minutes, and anonymous surveys, and the BC Harm Reduction website. For documents, we reviewed *How to Involve People Who Use Drugs (2014)* [19], the *BC Harm Reduction Strategies and Services Committee Terms of Reference (2012)* [15] and an internal document, *Peer Consultants at HRSS Face-2-Face (2014)*. Summary notes were available from focus groups and telephone interviews that had been conducted to inform a research funding application on peer engagement in BC (Peter Wall Solutions). Researchers at the BCCDC consulted with seven representatives from all BCHRSS member organizations and interviewed three groups of peers from two health regions in 2014. Conversations with representatives and peers were not transcribed thus preventing detailed secondary data analysis for this evaluation. However, a document summarizing key themes to inform current and future peer engagement efforts was available, in addition to the grant application itself, and incorporated.

A review of the literature revealed a lack of evaluation frameworks based on the principles of health equity, as well as frameworks for engagement with people who use drugs. *Public* engagement evaluation tools were limited in that most focused on outcomes, such as cost-effectiveness, rather than considering the process of engagement itself. The *Involve UK’s* guidelines for public participation in central government most closely resembled our aims and equity-based evaluation principles [20]; thus, we adapted it, broadly using the language, themes, and structure as a general guide to developing an evaluation framework specific to peer engagement [see Table 1]. The four goals identified for evaluation were supportive environment, equitable participation, capacity building and empowerment, and improved programming and policy. Examples of constructs were derived from the evaluation of evidence itself and are by no means exhaustive or inclusive.

**Table 1:** Peer engagement process evaluation framework

Goal	Assessment	Description	Examples of constructs
Supportive Environment	How were barriers and facilitators to engaging addressed?	Assess and address barriers and facilitators of engagement; ‘environment’ encompasses micro (i.e. power dynamics between individuals), meso (ie. organizing transportation to/from), and macro levels (i.e. meeting location).	• Easy access/low threshold meetings (immediate compensation, supportive arrangements for people travelling from out of town by paying transportation costs in advance) • Community building activities • Building, location chosen • Planning in advance • Flexible schedule
Equitable participation	How were experiences represented and respected?	Ensure all experiences respected and represented at the table to address the diverse and unique health needs of each community.	• Democratic participation • Power dynamics • Flexible facilitation • Distribution of voices • Representativeness at the table • Awareness of peer issues and strengths within the community
Capacity building & empowerment	How did capacity increase over time and how were benefits derived?	Develop the abilities of individuals and groups defined in terms of access, ability, mobilization, interest, networks, opportunity, and literacy.	• Skills and ability • Confidence • Ongoing engagement or attrition • Social capital • Community building • Enhanced peer networks • Cohesion
Improved programming & policy	How engagement impacts programming and policy?	The explicit and implicit evolution of programming and/or policy in relation to the purpose identified; ability to understand local risk environment, synthesize information, and design relevant solutions.	• Programming and/or policy • Competency • Activities • Outputs • Feedback from within and/or outside the inner and/or broader community

The evaluation framework allowed us to compare and contrast multiple sources of information and outcomes, increasing the validity of our findings through triangulation [21]. For example, we examined information about meetings involving peers by reviewing the agendas, minutes, attendee evaluation surveys, and post-meeting debriefing notes. Where sources provided similar data, we simplified and summarized the messages, and where sources provided different information, we presented the full breadth of perspectives. Our evaluation failed to present any contradictory information requiring resolution.

The University of British Columbia Research Ethics Board granted the ethics approval for the interview and focus group data used in this evaluation.

## Results

The evaluation results in Table 2 outlines our assessment of peer engagement processes in BC from 2010–2014. These results focus mainly on events surrounding peer engagement with the BCHRSS, but also involve several other harm reduction initiatives in BC at that time.

**Table 2:** Evaluation Results from the British Columbia Harm Reduction Programme: lessons learnt, evidence of progress and opportunities for improvement, 2011–2014

Construct	Lessons Learnt	Evidence of progress	Opportunities for improvement
GOAL:	Supportive environment	(How were barriers and facilitators to engaging addressed?	
Community Building activities	• Reported feelings of exclusion among peers • Lack of trust or legitimacy built early on members and other peers	• Introduced various team-building activities and ice breakers to build trust & openness • Included Aboriginal opening and closing ceremonies, and pre-meeting dinner social	Form peer advisory group that is engaged with HRSS committee throughout the year
Planning in advance	• Peers unaware of role and expectations; some informed of meeting with too short of notice	• Invited multiple peers at least six weeks in advance • Arrangements provided for transportation, accommodation, local support (i.e. methadone)	Develop list/map of commonly accessed resources in host community
Structure of Schedule	• Lack of opportunity to develop rapport and trust with committee • Inconsistency of information	• Agenda modified based on feedback provided by peers before, during and after meeting • Meeting agenda more flexible with less content	Develop agenda together (i.e. with peers and committee)
GOAL:	Equitable participation	(How were experiences represented and respected?)	
Representativeness at the table	• Unequal representation from health authorities due to staffing issues or lack of commitment from region	• Shifted to inviting two peers per health region • Caravan project traveled to rural regions to meet peers “where they’re at”	Form peer advisory group engaged with BCHRSS throughout the year
Power Dynamics; Distribution of voices	• Inequitable distribution of power among peer groups and across	• Provided peers with cash stipend based on wage • Extra attention paid to distribution of power, people at the table, voices being heard • Discussions captured on flipchart so peers could see their voices being heard and respected • Shorter duration of roundtable updates allowed time and space for peers to voice their concerns	Consider options for peers to communicate their thoughts in non-verbal ways or in smaller groups; routine check-ins with peers during breaks
Flexible Facilitation	• Heterogeneous representation of peers at the table • Rural/remote regions need attention	• Attention paid to the attitudes during activities; able to adapt based on energy/positivity in room • Kept discussion positive and solutions-based	Ongoing need for strong but flexible facilitator
GOAL:	Capacity building & empowerment	(How did capacity increase over time and how was it built on?)	
Community Building	• Lack of opportunities initiated outside the BCHRSS meetings • Staffing issues remain a problem	• Peer engagement activities supported financially through funds offered in each health authority • Beginning of peer-based harm reduction supply distribution & education	Develop sustained, ongoing funding mechanism e.g. for work contracted to peer organizations
Social Capital; skills &ability; confidence	• Inability to build on existing capacity within communities	• Peers create EIDGE group with illicit alcohol users • Peer groups organize around key issues: social housing, anti-harm reduction by-laws, methadone formulation change	Social capital is strongest in urban peer groups; knowledge transfer needed with rural peer groups
Enhanced Peer networks	• Efforts fragmented across province • Some drug user organizations dissolved due to lack of support	• Peer network in BC grows via BCHRSS meetings, HR activities; opportunities for growing peer-run orgs	Build organizational capacity to increase autonomy from any group of peers
GOAL:	Improved policy & programming	(How engagement impact programming and policy?)	
Improved harm reduction programming	• Identified inconsistent access to harm reduction supplies • Lack of capacity building and training for peer workers, service providers and decision makers	• The Caravan Project • Expanded range of supplies to include safer inhalation supplies • Introduced BC Take Home Naloxone program • Developed specialized harm reduction trainings; posted training manual online • Introduced annual harm reduction client survey	Budget and other organizational constraints limit the expansion of comprehensive harm reduction services – (frustrating for peers)
Improved policies	• Lack of peer engagement at other tables outside BCHRSS • Lack of best practices on best ways to engage peers	• Developed one-page guidelines for providers on inviting peers to meetings • Peer engagement literature review (Ti et al., 2012 [7]) • Improved documentation and dissemination of HRSS policies and research for lay audiences	Develop best practice guidelines for services to meaningfully engage peers
Activities	• No formal process or evaluation of peer engagement in BC • Inconsistent effort to implement processes, sustain initiatives	• Obtained financial support for peer engagement research in BC • Presented results and reports on peer engagement to stakeholders across the province	Evaluate best practice guidelines to ensure acceptability in different contexts (regions, populations)

### Supportive environment

**Fig. 1:**
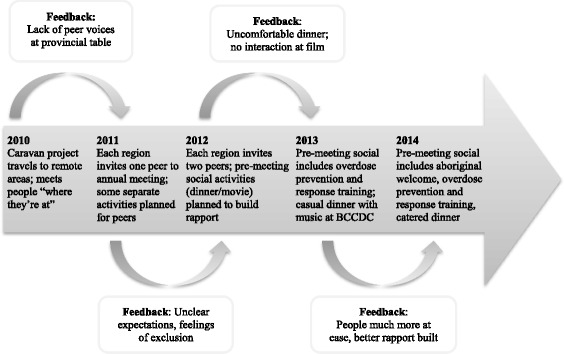
Evolution of the equitable and enabling environment of peer engagement 2011–2014

Peer engagement in BC was an iterative process that increased and improved over time as consequence of reflexive learning. When the committee began inviting peers to meetings, peers were not given clear expectations. Feedback revealed planning in advance, making travel arrangements, setting up support locally, and creating a welcoming environment were important factors for supporting and facilitating engagement. Organizing local methadone prescriptions or making arrangements for connecting to local peers for other supplies well in advance was essential for out-of-town peers to feel supported. It also contributed to their ability to be fully present at meetings. Sending an agenda and/or itinerary in advance developed peers expectations for the meeting. To establish rapport between attendees prior to the meeting, community-building activities were organized. Dinner at a restaurant was not comfortable, but in subsequent years sharing a meal in a more relaxed setting worked well. Developing a peer advisory group who could provide feedback on the meeting agenda and other activities was one way to improve on existing efforts. Figure 1 is one example of the equitability and environment of peer engagement in BC, offering a visual representation to highlight this reflexive and cyclical process.

### Equitable participation

In the beginning a single peer was invited to meetings. Activities offered to peers were separate from the rest of the committee. Feedback about the need for peer support led the committee to extend its invitation to two peers per region starting in 2011. Being in a new and/or different place can be destabilizing for some, leading to “triggering” or inducing behaviors peers would otherwise try to avoid. As such, offering supports around the meeting was imperative to equitable participation especially when working with a population with diverse needs and backgrounds. Due to the geographic structure of health authorities in BC, there was an unequal representation of peers from different regions at the meetings. Peer run organizations or groups, such as VANDU in Vancouver Coastal Health Authority, were asked to nominate participants as representatives from their regions. In areas where organized peer groups did not exist, such as the Northern Health Authority, harm reduction coordinators in were encouraged to invite peers who they had worked with during the year and to use the meeting as an opportunity to promote sustained involvement. Peers who had transportation, disability or mobility issues were accommodated. Nonetheless, peers from rural regions remained underrepresented, likely due to the geography and inconsistent staffing in these regions (i.e. Northern Health Authority, Interior Health Authority). Providers and peers from these regions were expected to travel great distances (sometimes more than 500 kilometres) to engage or reach services, greatly limiting engagement opportunities. “One-off” or one-time engagement opportunities outside of the BCHRSS were common but fragmented across events. Ongoing opportunities and strategies to keep peers engaged in the long run were needed. Peers also drew attention to power imbalances. Being attentive to the distribution of voices at the table so everyone was treated equally and respectfully was important. The need for strong but flexible facilitation, check-ins, and options to share ideas anonymously were identified as opportunities for improvement.

### Capacity building and empowerment

The BCHRSS committee supported the expansion of peer networks through research, networking, and funding opportunities. In 2010, the BCHRSS committee supported the BC-Yukon Association of Drug War Survivors to drive across BC as a team, meeting peers “where they’re at,” conducting harm reduction workshops and gathering information about peers’ health needs. This project, known as the “Caravan Project,” [22] highlighted the informal peer engagement efforts to date, and served as the impetus to examine and enhance peer engagement in BC. Peers from various regions were inspired by the bold leadership of representatives from peer-run organizations, such as the Society of Living Illicit Drug Users (SOLID) and VANDU, and some became more involved in peer activities in their own communities. Some efforts were financially supported by BCHRSS funds to form new entities, such as the Kelowna Area Network of Drug Users (KANDU). From this project, the Eastside Illicit Drinkers Group formed.

Even with limited financial supports, peer networks across BC were able to mobilize around important issues, such as social housing, anti-harm reduction by-laws, and methadone formulation changes. In 2013, the SOLID organized and hosted a national symposium of peers in Victoria that kick-started a conversation nationally with 14 peer-run organizations. These organizations documented their successes for other peer-run organizations and allies to learn from by creating a document called “Collective voices for Effective Change” [13]. We noted that the ability to distribute this information, form new peer networks, and build on already established capacity among groups of peers was limited due to financial and geographic challenges.

### Policy and programming

The BCCDC provided resources for the “Caravan Project,” [22] which identified the need for several policy and program initiatives through focus groups with peers across BC. Eight priority areas for promoting health equity were offered. In 2011, the BCCDC implemented two policy changes as a direct result of these findings: 1) regional representatives were asked to invite a local peer to the face-to-face meeting held in the spring of 2012, and 2) annual funds ($2000) were offered to support peer-led initiatives for harm reduction activities and matching funds ($5000) for community development activities in each health authority [15]. Peer engagement opportunities and feedback on policy and programming increased as a result, which had additional effects beyond the BCCDC. Harm reduction sites started providing safer inhalation supplies and the BC Take Home Naloxone program was launched. Also, an annual client survey at harm reduction supply distribution sites was introduced.

Despite increased support for peer engagement by the BCHRSS, the lack of a formal peer engagement processes or guide as to *how* stakeholders reach out to engage peers, including how to invite, involve, and encourage participation, led to inconsistent efforts. The committee struggled through staffing changes and turnover, leading to disjointed peer engagement efforts. These internal gaps contributed to the disbanding of some independent peer networks (i.e. KANDU) and inconsistent support for new networks.

## Discussion

This manuscript shares the lessons learned by the BCHRSS committee in adopting peer engagement practices in harm reduction initiatives in BC from 2010–2014. Increasing capacity and equity of peer engagement, as well as positive program and policy changes were evident throughout. We found providing clear expectations of the roles of peers and committee members at meetings and purposeful engagement opportunities influenced the quality and overall success of events. However, organizational constraints, including staff and peer turnover, were ongoing issues in terms of achieving opportunities for equitable peer engagement. Where there was turnover, it was imperative new staff and peers are informed of previous practices, discussions, and cultural context. We also found that geography was a persistent challenge for peer engagement in BC. For instance, Northern Health Authority spans over half the province geographically, yet has the smallest population. The health needs in rural areas have been found to differ from those in urban areas, therefore requiring “rurally sensitive” initiatives [23].

This evaluation provides a case study of the cyclical and iterative nature of peer engagement. Public participation literature highlights the interrelated, iterative steps in the engagement process, as a cycle of 1) designing for context; 2) enlisting and managing resources; and 3) evaluating and redesigning continuously [24]. Given that peer engagement is relatively new both locally and internationally, learning from past successes and failures is key to developing effective initiatives [24]. To ensure the integrity of this cycle, we learned and stress the importance of unwavering commitment to this work, both in terms of financial and staff resources, tailored to the context and individual experiences that vary among peers.

A large body of evidence exists that supports citizen engagement in policy and program decisions to more effectively address the needs of the public. Some argue participation may be ineffective and costly [25], while others see the public as the “most important stakeholder in the health care system” [26]. In a review of the literature, Marshall et al. suggests systemic, organizational, and individual obstacles to peer roles exist in harm reduction initiatives, including stigmatization, inadequate training, and lack of availability of peer roles [7]. It may be that the effectiveness of engaging and involving peers depends on methods adapted to population and context [27]. Literature stresses that both the *form* of engagement as well as the interactions that *build trust and legitimacy* promotes meaningful and sustainable relationships between stakeholders [8, 24]. Establishing legitimacy may be particularly important for marginalized groups. Therefore, practical ways to develop trust, redress power imbalances, and improve relationships must be continually assessed and addressed [26]. Marginalized groups face unique interpersonal and structural obstacles that may restrict their involvement in decision-making [7, 8, 16]. Similarly, by excluding some of the most marginalized members of society, we risk exacerbating disparities among these groups [26].

Support for engaging peers at the decision-making table has been expanding both locally and internationally [26]. Within Canada, peer input has influenced decisions around harm reduction best practices [6], [28] and messaging for overdose prevention [29]. Examples such as these are evidence that peer engagement has the potential to advance social justice by improving equity in the distribution of services or by increasing marginalized groups’ influence over decisions [24, 30]. Although there has been increasing evidence of positive outcomes from peer-run programs, attention paid to upstream policy and program development is still lacking [7], [8]. During this process evaluation, a time of increasing efforts to integrate peer engagement in harm reduction services and strategies in BC, peer groups across Canada noted the lack of initiatives for meaningful peer involvement in policies and programs across the country [13].

Although we developed our own peer engagement process evaluation framework, efforts remain difficult to measure and evaluate. There is no single set of metrics in any evaluation of public engagement, but rather a subset of criteria based on most desirable implementation outcomes [24]. Our evaluation framework focused on four engagement goals that are not mutually exclusive and non-exhaustive; other aspects to peer engagement may need additional evaluation. The public health outcomes of peer engagement in harm reduction were not examined as they were outside of the scope of this research. As well, interactions between service providers and peers were not examined but likely played a key role in the success of engagement efforts. The community and relationships built through peer engagement are also beneficial products of this process. However, for the purposes of this evaluation, these factors were not examined. Future research should examine interpersonal factors that are at play in peer engagement in harm reduction and the potential impact peer engagement can have on overall public health. Any unintended consequences from any peer engagement approach, such as tokenistic engagement opportunities, “triggering” or destabilizing situations, or exploitation of peers, should also be explored and documented in greater detail. Procedures offered in this evaluation may be used as strategies to avoid some negative consequences of peer engagement.

Furthermore, peer engagement is an evolving process, influenced by many external ecological factors over time. It may not be possible to measure latent impacts of policy choices or peer engagement within the timeframe of the evaluation itself [24]. It is difficult to attribute findings to one decision or time as engagement evolves within an ever-changing context. Most data was qualitative, requiring a subjective, retrospective assessment of quality, rather than a quantitative indicator of success or failure. There are likely several other aspects to peer engagement that were not evident, such as improving health and social networks. Given that consultations with peers and harm reduction representatives were intended to identify and inform the application of peer engagement processes, we did not record and transcribe these conversations. The retroactive assessment of peer engagement and lack of primary data from peers in a major methodological limitation of this evaluation. The voices and experiences of peers and harm reduction representatives are crucial to improving future peer engagement initiatives. It is our hope that future research will consider more in-depth experiences of peer engagement to provide a richer understanding of peer engagement processes and practices in the field.

## Conclusions

Overall, this evaluation emphasized the ongoing importance to engage peers in the planning, delivery, and evaluation of harm reduction initiatives. It provided a foundation for establishing new or promoting existing peer engagement. Broadly, this evaluation offers some of the first evidence showing peer engagement as a tool for policy change, capacity building, and equity by facilitating inclusion regardless of social position or other circumstance. Findings included important lessons learned and strategies for improving the implementation, delivery, and sustainability of peer engagement. Several practice recommendations based on crosscutting themes were highlighted in our evaluation, derived for designing future peer engagement initiatives [see Table 3]. Finally, we offer opportunity to future research to further develop our evaluation framework for peer engagement in other health equity contexts.

**Table 3:** Recommendations for peer engagement in harm reduction initiatives

• Create a low barrier, low threshold environment adapted to the context of the peers involved
• Use reflexivity, reflecting and learning from the process
• Define roles and expectations for all stakeholders
• Be conscientious of who is at the table and prioritise traditionally under-represented peer groups (e.g. those from rural and remote communities)
• Develop formal best practice peer engagement guidelines
• Ensure consistency across regions and stakeholders
• Provide support for building and connecting new and existing peer networks
• Make the most of and expand on capacity that has already been built
• Promote ongoing commitment to the process from all stakeholders

## Abbreviations

BC, British Columbia; BCCDC, British Columbia Centre for Disease Control; BCHRSS, British Columbia Harm Reduction Services and Strategies; KANDU, Kelowna Area Network of Drug Users; SOLID, Society of Living Illicit Drug Users; VANDU, Vancouver Area Network of Drug Users
